# Mediterranean Journal of Rheumatology December 2018 Highlights

**DOI:** 10.31138/mjr.29.4.182

**Published:** 2018-12-18

**Authors:** Theodoros Dimitroulas

**Affiliations:** Aristotle University of Thessaloniki, Thessaloniki, Greece

In this December’s issue, the readers of MJR will find interesting papers including reviews, original articles and case reports, as well as a number of externally reviewed research protocols approved for funding by the Greek Rheumatology Association (ERE).

In their editorial, Gasparyan and Kitas^1^ address open access publishing and necessary steps to ensure its quality, highlighting important tools and effective strategies. Next, enteric microbiota is considered as one of the important triggers of autoimmune activation in rheumatic diseases. Bogdanos and Sakkas^2^ discuss the potential involvement of *Enterococcus gallinarum*, a Gram-positive gut pathobiont, in the pathogenesis of autoimmunity with a special focus on the co-existence of systemic lupus erythematosus and autoimmune hepatitis. Based on preliminary data form animal models and humans, the authors hypothesize that *Enterococcus gallinarum*, may represent a mechanism which mediates immune dysregulation particularly in the gut-liver axis in lupus patients with further evidence required from liver biopsies to check for bacterium translocation.

Dr Argyropoulou and Prof Tzioufas^4^ – one of the leading international experts in Sjögren’s Syndrome – provide an excellent update of the disease for consultants and rheumatology registrars covering the pathophysiological, clinical and therapeutic aspects of the disease with important implications for daily clinical practice.

The important role of exercise as medicine in several aspects of the management of rheumatoid arthritis such as control of inflammation, improvement of functional status and reduction of cardiovascular risk has been established over the last few years despite the lack of specific recommendations – at least for the time being - by EULAR or ACR. Weijers et al.^5^ provide data from two Dutch rheumatoid arthritis cohorts, demonstrating that the level of physical activity between 2013 and 2016 appears to have increased, with patients spending more minutes per week in exercise activities. The authors also discuss methods of motivating patients to increase physical activity, such as tailored exercise programs with a focus on setting personal goals different for each individual. It would be interesting to find out whether similar trends have occurred in other countries and whether the magnitude of the increase is sufficient to confer health benefits. Better understanding of the autoinflammatory diseases has resulted in earlier diagnosis and improved treatment strategies. In this issue, Alzyoud et al.^7^ present their experience from Jordan in children diagnosed with familiar Mediterranean fever. Given that the disease is far more common in the Middle East, such reports contribute to the dissemination of medical information and give the opportunity to learn from each other’s experience across different countries.

Functional disability driven by knee osteoarthritis is a major issue for society and health systems. In that respect, Gorial et al.^6^ have investigated the association between functional status and demographic and clinical parameters in Iraqi patients. In line with previous reports, they demonstrated a close relationship between the functional burden of the disease with age and severity of osteoarthritis, and they confirm an unfavorable impact of low educational level in the course of the disease.

Systemic lupus erythematosus remains a challenge in rheumatology practice, and the management of the disease becomes more complex in case of uncommon and/or under-recognized side effects of immunosuppressive treatment. As the routine administration of mycophenolate mofetil is expanding across the whole spectrum of disease manifestations, unknown side effects are emerging; their knowledge is highly relevant for clinicians. For example, new-onset acne in lupus patients receiving mycophenolate mofetil may be related with the treatment, as suggested by the small case-series study by Perricone et al.^8^ In addition to describing the cases, the authors also suggest treatment options that may prove useful in routine clinical practice.

In this issue, Antonopoulos et al.^9^ describe an interesting case of cutaneous dermatomyositis refractory to therapy with conventional and biologic disease modifying drugs, which finally improved with intravenous immunoglobulin. Such cases underline the complexity of patients with inflammatory myopathies and emphasize the necessity for off-label treatment approaches in specific patients.

MJR hosts research protocols funded – after international peer-review and validation process - by the Greek Rheumatology Society and Professional Association of Rheumatologists (ERE-EPERE). These research projects cover a broad spectrum of basic, clinical and epidemiological studies. This round includes several particularly interesting projects. Skarlis et al.^10^ will assess the prevalence of osteoporosis in Sjögren’s syndrome and investigate the activation of the RANKL / RANK and osteoprotegerin system in peripheral blood and salivary gland biopsies of patients and controls. Adamichou et al.^12^ will compare the sensitivity and specificity of the earlier and new classification criteria for systemic lupus erythematosus in a retrospective study, and also examine how the criteria perform regarding the earliest classification of lupus in a prospective cohort of patients with undifferentiated connective tissue disease. Karageorgas et al.^13^ set up an epidemiological multicentre study to assess the clinical characteristics and outcomes in patients with rheumatoid arthritis and interstitial lung disease in Greece. Last but not least, Ntali et al.^11^ present the outline of the Birth Registry of Greek women with systemic lupus erythematosus which will provide extremely useful information for this group of patients in whom pregnancy is considered of “high-risk” for several reasons including relapse of the disease, concomitant treatment and complication for the mother and the foetus.

Finally, Dr Nikas, the special secretary of ERE-EPERE, summarises the highlights of the recent 26^th^ Panhellenic Congress of Rheumatology^3^ that took place in Athens, Greece on 6–9^th^ December 2018.

We would also like to use this opportunity to express again our sincere gratitude to the reviewers - listed in the current issue - who provided their judgment concerning the quality of the submitted papers and have enormously contributed to the progress of *MJR* in 2018.
